# *MEG8* as an antagonistic pleiotropic mechanism in breast cancer

**DOI:** 10.1038/s41420-024-02272-0

**Published:** 2024-12-20

**Authors:** Eva M. Verdugo-Sivianes, Asunción Espinosa-Sánchez, Ildefonso Cases, Ana M. Rojas, Daniel Otero-Albiol, Lourdes Romero, José Ramón Blanco, Amancio Carnero

**Affiliations:** 1 Instituto de Biomedicina de Sevilla (IBIS), Hospital Universitario Virgen del Rocío (HUVR), Consejo Superior de Investigaciones Científicas, Universidad de Sevilla, Seville, 41013 Spain; 2 CIBERONC, Instituto de Salud Carlos III, Madrid, 28029 Spain; 3 Centro Andaluz de Biología del Desarrollo (CABD), CSIC-Universidad Pablo de Olavide, Sevilla, Spain; 4 Hospital Universitario San Pedro, 26006 Logroño, Spain; 5 Centro de Investigación Biomédica de La Rioja (CIBIR), 26006 Logroño, Spain; 6 Present Address: Departamento de Ciencias de la Salud y Biomédicas, Facultad de Ciencias de la Salud, Universidad Loyola Andalucía, Avda. de las Universidades s/n, 41704 Dos Hermanas, Sevilla Spain

**Keywords:** Senescence, Breast cancer, Breast cancer

## Abstract

Cellular senescence connects aging and cancer. Cellular senescence is a common program activated by cells in response to various types of stress. During this process, cells lose their proliferative capacity and undergo distinct morphological and metabolic changes. Senescence itself constitutes a tumor suppression mechanism and plays a significant role in organismal aging by promoting chronic inflammation. Additionally, age is one of the major risk factors for developing breast cancer. Therefore, while senescence can suppress tumor development early in life, it can also lead to an aging process that drives the development of age-related pathologies, suggesting an antagonistic pleiotropic effect. In this work, we identified Rian/MEG8 as a potential biomarker connecting aging and breast cancer for the first time. We found that Rian/MEG8 expression decreases with age; however, it is high in mice that age prematurely. We also observed decreased MEG8 expression in breast tumors compared to normal tissue. Furthermore, MEG8 overexpression reduced the proliferative and stemness properties of breast cancer cells both in vitro and in vivo by activating apoptosis. MEG8 could exemplify the antagonistic pleiotropic theory, where senescence is beneficial early in life as a tumor suppression mechanism due to increased MEG8, resulting in fewer breast tumors at an early age. Conversely, this effect could be detrimental later in life due to aging and cancer, when MEG8 is reduced and loses its tumor-suppressive role.

## Introduction

Aging is characterized by the deterioration of an organism’s functional capacities in a progressive and generalized form, resulting in age-associated pathologies and more difficult adaptation to the environment [1]. The relevant aging features are gradual loss of function or degeneration at the molecular, cellular and tissue levels, the changes that allow cells to proliferate inappropriately [2]. Therefore, aging is considered an important risk factor for developing many diseases. These diseases can be classified into two categories: loss-of-function diseases, such as neurodegenerative, cardiovascular, or osteoporosis, and or diseases through gain-of-function, which are generally hyperplastic pathologies, the most common of which is cancer [3]. These two types of age-related diseases are partly connected by a common biological mechanism: cellular senescence [2].

Cellular senescence is activated by normal cells to respond to cellular stress, such as DNA damage, oxidative stress, telomere shortening, or unrequired oncogene stimuli [3, 4]. In this cellular stress response, cells exit the cycle, losing the ability to proliferate by mitogenic stimulation, and undergo several morphological and metabolic changes [3–5]. Therefore, senescence is a tumor-suppressive mechanism that promotes tissue repair or regeneration, a process that malignant tumor cells need to bypass [2, 3]. Cellular senescence, therefore, plays an important role not only in tumor suppression but also in aging by promoting chronic inflammation [2, 4, 5]. It is thought that the regeneration of somatic tissues provokes the accumulation of senescent cells, limiting tissue renewal, disrupting homeostasis of normal tissues, and ultimately contributing to aging. Furthermore, both senescence and aging are related to telomere shortening and limit lifespan [5].

Cellular senescence adheres to the antagonistic pleiotropy theory, as it suppresses cancer development early in life but drives age-related pathologies later in life. In addition, one of the most significant risk factors for developing cancer is age, and age-related deterioration of the organism alters the senescence response, creating a permissive tissue microenvironment that allows the development or progression of cancer [1–3].

Aging, cancer, and cellular senescence are undoubtedly connected, but the mechanisms responsible for this connection are not completely understood. Although it seems likely that people age at different rates, there is no precise way to quantify aging other than chronological age, making it necessary to better quantify aging. Genotypes that reduce life expectancy are likely to increase the risk or severity of age-related diseases [6, 7]. Indeed, genes involved in longevity or delayed aging are inversely related to genes associated with cancer [8, 9]. Breast cancer is the most common neoplasm and the main cause of cancer-related death in women worldwide [10–12]. Several risk factors increase the probability of breast cancer, one of which is age over 50 years [13]. Additionally, breast tumors are highly diverse and complex, as they present different molecular, physiological, and morphological characteristics, resulting in varied responses to conventional treatment [14]. The diversity of these tumors needs the search for new predictive and prognostic biomarkers and new therapeutic targets to improve cancer treatments [15].

In this work, we used three different strains of mice with different lifespans to study the relationship between aging and cellular senescence. We used normal C57BL/6 J mice and prematurely aged mice (SAMP6/TaHsd or AKR/J strains) to compare the expression profile in breast tissue via microarray analysis. The results showed that prematurely aged mice expressed higher levels of the Rian gene than C57BL/6 J mice in breast tissue, and all types of mice showed a decrease in Rian expression levels with age.

Rian (RNA imprinted and accumulated in nucleus) is a mouse long noncoding RNA (lncRNA), and its human ortholog is MEG8 (Maternally expressed 8). Rian/MEG8 is located in the DLK1-DIO3 cluster, which contains several coding genes expressed from the paternally inherited chromosome (DLK1, RTL1, and DIO3), as well as several large and small imprinted noncoding RNA genes from the inherited maternal homolog, including Rian/MEG8 [16]. Moreover, the Rian/MEG8 gene can be transcribed into different long and short noncoding RNAs, including both miRNAs and snoRNAs [17, 18]. However, the role of Rian/MEG8 in aging and cellular senescence remains largely unknown. In this work, we explored the role of Rian (RNA imprinted and accumulated in nucleus)/MEG8 in these processes to identify the connection between aging, cellular senescence, and cancer in breast tissue.

## Results

### *Rian/MEG8* levels decrease with age and are higher in mice that age prematurely

To study aging in mice, we used different strains of mice that allow us to compare aging in normal C57BL/6 J mice with premature aged mice (SAMP6/TaHsd or AKRJ strains). SAMP6 (Senescence Accelerated Mouse Prone 6) mice show accelerated aging due to a senile osteoporosis characterized by a diminished bone formation and a paucity of osteoblast progenitor cells [19] whereas AKRJ mice present a high leukemia incidence (60-90%) [20]. The lifespan of C57BL/6 J mice is about 900 days, whereas SAMP6 or AKRJ mice have a lifespan of less than 300 days [19, 20]. We keep the three different strains of mice in identical controlled conditions and we sacrificed them at 3-4 weeks or 16 weeks and at 93 weeks in the case of C57BL/6 J mice, selecting three females and three males of each condition. We removed breast tissue from all mice, and we performed a microarray analysis.

**Fig. 1:**
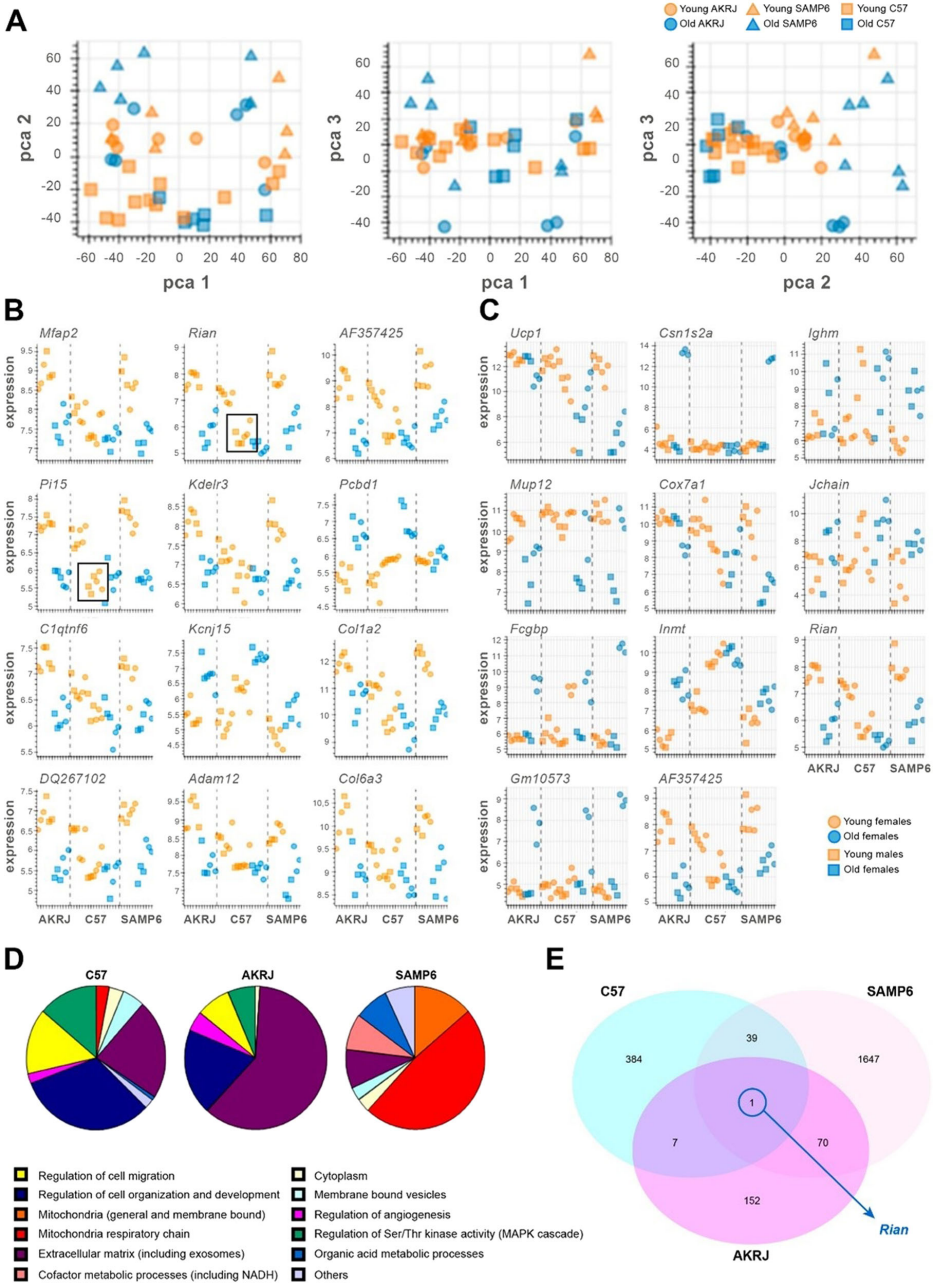
*Rian* is the only gene that had a different expression pattern in the breast tissue of mice that age differently. **A** PCA analysis of the 42 samples of the microarray. Young (orange) and old (blue) samples of the three strains of mice (circles for AKRJ, triangles for SAMP6, and squares for C57) were compared. **B**, **C** Differentially expressed genes of young (orange) and old (mice) of the three strains. Sex is represented in circles (females) or squares (males). We included the most significant changes (**B**) and the most pronounced changes (**C**). **D** Functional enrichment analysis of the differentially expressed genes of the three strains. **E** Venn diagram showing the differentially expressed genes of the three strains.

First, by a PCA analysis, we compared all the samples of the three strains of mice, and we found that the samples do not show variation between them. We compare young versus old mice, but they do not show variations between old/young phenotypes, so any observation is not biased (Fig. 1A). Then, we performed an analysis of old versus young variations by strain to find those genes differentially expressed between young and old mice in each strain. We found that C57 mice of 16 weeks seem to be as older as the other two strains. We also observed that the genes that showed the most significant changes were *Rian* and *Pi15* (Fig. 1B). Indeed, *Rian* was included in the genes that showed the most pronounced changes (Fig. 1C). Then, we performed a functional enrichment analysis to observe the gene ontology and the KEGG pathways of those genes differentially expressed. We found that the pathways of C57 and AKRJ mice were very similar, and the majority of these genes belonged to extracellular matrix pathways, regulation of cell organization and development pathways, regulation of cell migration pathways and regulation of kinase activity pathways, among others. In the case of SAMP6 mice, most of the genes belonged to pathways related to the mitochondria respiratory chain and mitochondria in general, but they also belonged to extracellular matrix pathways and other metabolic processes (Fig. 1D). Next, we performed a Venn diagram to show the overlap of the differentially expressed genes of the three strains including old and young mice and we only find one gene: *Rian* (Fig. 1E). Therefore, the microarray analysis showed that *Rian* is the only gene that had a different expression pattern in breast tissue in the three strains of mice.

**Fig. 2:**
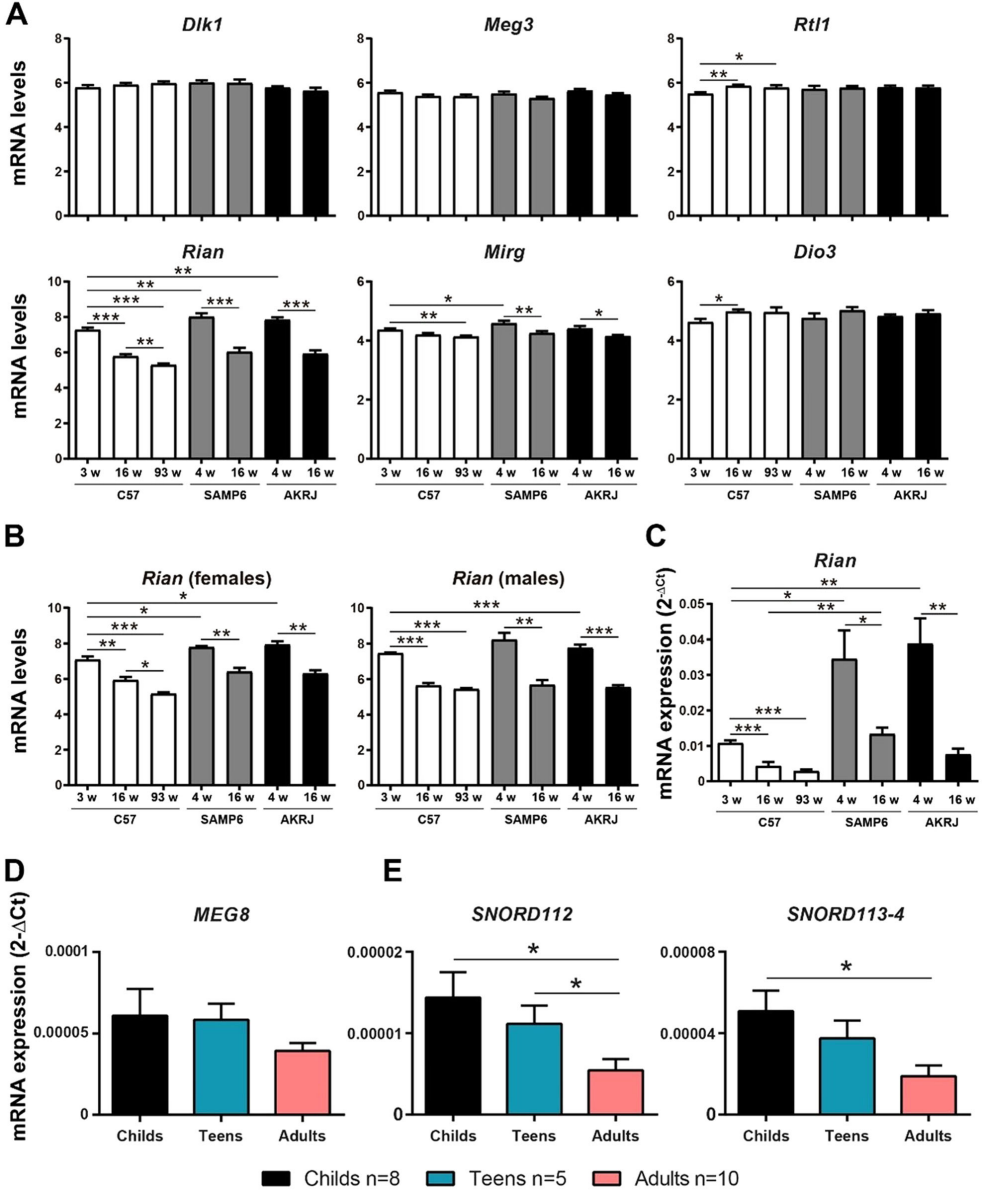
*Rian/MEG8* expression decreases with age. **A** Expression levels of the Dlk1-Dio3 cluster genes by a microarray analysis in the breast tissue of three different strains of mice, C57BL/6 J, SAMP6 and AKRJ. We used 3/4-, 16- or 93-weeks (w) old animals, selecting three females and three males of each condition. **B** Expression levels of *Rian* in the microarray analysis. Here, we independently show the results of females and males (n = 3 in each condition). **C** Measurement of *Rian* expression levels by RT-qPCR using the same samples of the microarray. **D**, **E** Measurement of *MEG8/SNORD112/SNORD113-4* expression levels by RT-qPCR using human blood samples from a cohort of patients with different age. Data were analyzed using Student’s t-test. *p < 0.05, ** *p* < 0.01, *** *p* < 0.001.

*Rian* is a gene that can be transcribed into different long and shorts ncRNAs, snoRNAs and miRNAs (Fig. Supplementary 1A). *Rian* is part of the DLK1-DIO3 locus, which contains protein-coding genes, *DLK1*, *RTL1* and *DIO3*, expressed from the paternally inherited chromosome and several imprinted large and sncRNAs expressed from maternally inherited homolog, including *Rian* (Fig. Supplementary 1B). We analysed the expression levels of *Rian* and other genes of the DLK1-DIO3 cluster in the microarray and we did not observed difference in expression between the different breast tissue samples in these genes, except from *Rian* and *Mirg*, being *Rian* the gene more differently expressed between samples (Fig. 2A). We analysed separately females and males and we found that the pattern of expression of *Rian* is similar in both sexes, suggesting that it is independent on the sex (Fig. 2B). We also observed that the expression levels of the progesterone receptor (*Pgr*) and *Her2* genes increased with age in the three different strains, especially in females, whereas the expression levels of the estrogen receptor *Esr1* only increased in normal females (Fig. Supplementary S2).

Then, we validated this result by RT-qPCR, corroborating that *Rian* expression levels were higher in breast tissues of premature aged mice than in C57 mice. Furthermore, we observed that *Rian* expression levels decreases with age in breast tissue in the three strains of mice (Fig. 2C). Next, we analyzed the expression pattern of *MEG8*, the homologous of *Rian* in humans, in human blood samples of a cohort of patients with different age. We observed that adult patients presented lower expression levels of *MEG8* in blood samples than teen or child patients, demonstrating that *MEG8* expression also decreases with age (Fig. 2D). We also measured the expression levels of two different C/D box snoRNAs (SNORDs), *SNORD112* and *SNORD113*, that are located very close to *MEG8*. We observed similar results since the expression levels of *SNORD112* and *SNORD113* decreased with age, suggesting that they may be involved in aging as well (Fig. 2E). Therefore, *Rian/MEG8* levels decrease with age.

### The overexpression of *MEG8* induces senescence in normal cells

**Fig. 3:**
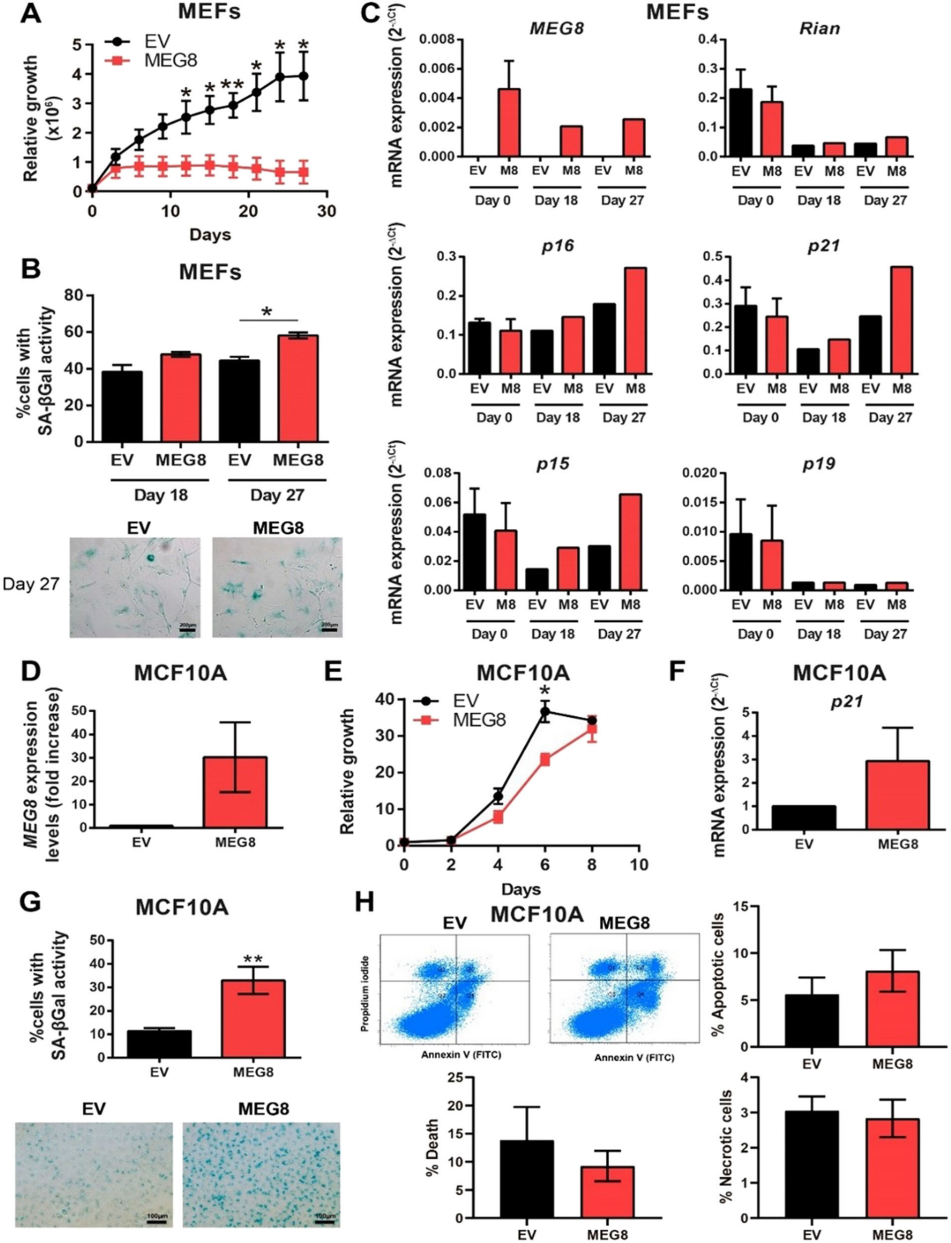
Overexpression of *MEG8* induces senescence in normal cells. **A** Growth curve of control and *MEG8* overexpressing mouse embryonic fibroblasts (MEFs). **B** Percentage of MEFs with senescence associated (SA) – β-galactosidase activity after Xgal staining. Representative images are shown. **C** Measurement of *MEG8, Rian, p16, p21, p15 and p19* expression levels by RT-qPCR in control and *MEG8* overexpressing MEFs. **D** Validation of *MEG8* overexpression in MCF10A cell line by RT-qPCR. **E** Growth curve of MCF10A control and *MEG8* cell lines. **F** Measurement of *p21* expression levels by RT-qPCR in MCF10A control and *MEG8* cell lines. **G** Percentage of MCF10A cells with senescence associated (SA) – β-galactosidase activity after Xgal staining. Representative images are shown. **H** Measurement of apoptosis in MCF10A control and *MEG*8 cell lines through FACS by using Annexin V and propidium iodide stainnings. We consider early-apoptotic cells (Annexin positive/propidium iodide negative cells), necrotic cells (Annexin negative/propidium iodide positive cells), and apoptotic cells (Annexin positive/propidium iodide positive cells). All the figures show the average of three independent experiments performed in triplicate. Data were analyzed using Student’s t-test. *p < 0.05; **p < 0.01; ***p < 0.001.

We have identified *Rian/MEG8* as a possible biomarker related to aging and senescence. Next, we explored the effect of the overexpression of *MEG8* in normal cells capable of enter in senescence. Therefore, we overexpressed *MEG8* and an empty vector (EV) as a control in mouse embryonic fibroblasts (MEFs) and then we followed the growth and appearance of cellular senescence features by performing a 3T3 protocol. We observed that those MEFs that overexpress *MEG8* enter in senescence earlier than control MEFs (Fig. 3A), showing a senescent morphological phenotype and a higher acid β-galactosidase activity, especially at day 27 (Fig. 3B). We validated the overexpression of *MEG8* by RT-qPCR and we observed that the levels of *Rian* did not change, so that overexpression was specific to *MEG8* (Fig. 3C). In addition, we observed an increase in the expression levels of some genes related to senescence, such as *p16INK4a, p21WAF1, p15INK4b* and *p19ARF*, in the cells that overexpress *MEG8*, especially at day 27 (Fig. 3C). Since we identified *Rian/MEG8* in breast tissue samples, then we studied the overexpression of *MEG8* in MCF10A [21], an non-tumorigenic cell line an immortalized from epithelial breast tissue. We validated the overexpression of *MEG8* by RT-qPCR (Fig. 3D) and we observed that MCF10A cells with increased *MEG8* grew slower than MCF10A control cells (Fig. 3E). Indeed, MCF10A-*MEG8* cells showed a high acid β-galactosidase activity (Fig. 3G) and increased levels of the senescence marker *p21WAF1* (Fig. 3F). On the other hand, MCF10A cells that overexpress *MEG8* showed no evidence of increased cell death, neither apoptosis nor necrosis (Fig. 3H and Fig. Supplementary S3). Therefore, it seems that the overexpression of *MEG8* induces senescence in non-tumoral MCF10A cells.

### The overexpression of *MEG8* decreases the proliferative capacity of breast cancer cells

**Fig. 4:**
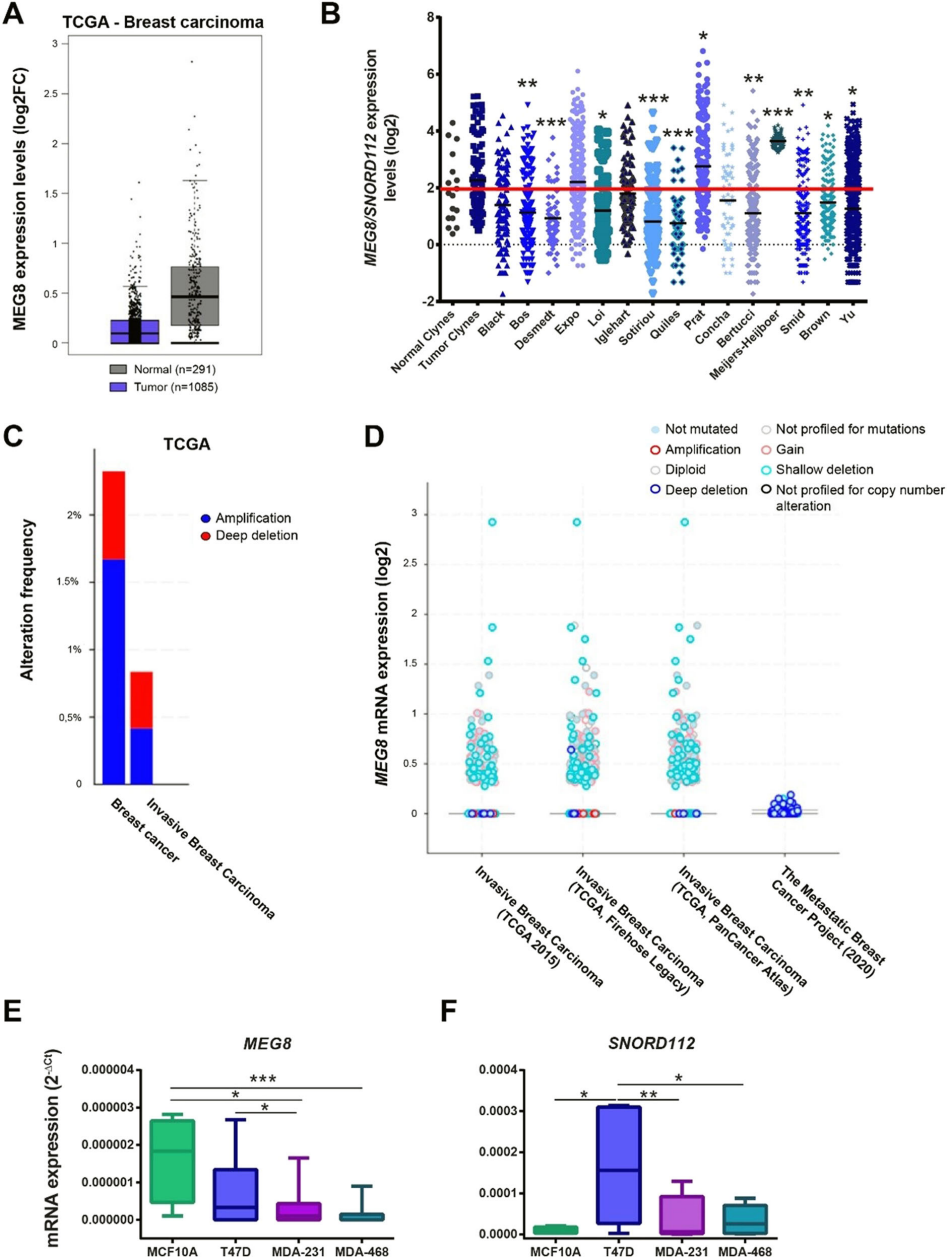
Decreased expression of *MEG8* in breast tumors. **A** Boxplot of *MEG8* expression in normal (black) and tumoral (blue) breast samples of patients with breast carcinoma from TCGA database. **B** Boxplot of *MEG8* and *SNORD112* expression in normal (black) and tumoral (blue) breast samples of patients with breast carcinoma from different breast tumor databases. Data were analyzed comparing tumor versus normal samples using Student’s t-test. **C**, **D** Alteration frequency of *MEG8* in breast samples of patients with breast carcinoma from TCGA database. Measurement of *MEG8* (**E**) and *SNORD112* (**F**) expression levels by RT-qPCR in the four breast cell lines T47D, MDA-MB-468, MDA-MB-231 and MCF10A. Data were analyzed using Student’s t-test. *p < 0.05; **p < 0.01; ***p < 0.001.

Then, we explored if there could be a relationship between aging of breast tissues and breast cancer. For that purpose, we analysed the levels of *MEG8* in patients with breast carcinoma from the database TCGA and we observed that breast tumor samples expressed lower levels of *MEG8* than normal breast samples (Fig. 4A). Additionally, we analysed the levels of *MEG8* and *SNORD112* in patients from different breast cancer databases and we found that in most of the databases the mean of *MEG8/SNORD112* expression of patients was lower than the mean of expression of the normal breast tissue database (Fig. 4B). Indeed, we also observed that breast tumors presented a higher proportion of shallow or deep deletions in the gene *MEG8* than the proportion of gains or amplifications (Fig. 4C, D).

Next, we studied the expression levels of *MEG8* and *SNORD112* in a panel of three different breast cancer cell lines: T47D, MDA-MB-468 and MDA-MB-231; and we also include the non-tumorigenic cell line of epithelial breast tissue MCF10A. We found that *MEG8* expression was higher in MCF10A in comparison with the three breast cancer cell lines and that this expression was lower in MDA-MB-468 and MDA-MB-231, two cell lines from basal/triple negative tumors, than in T47D, a luminal cell line (Fig. 4E). Therefore, it seems that *MEG8* expression decreased as the cell line was more tumorigenic or aggressive. In addition, we observed a similar behaviour in *SNORD112* expression, except from MCF10A cell line, whose levels were very low (Fig. 4F).

**Fig. 5:**
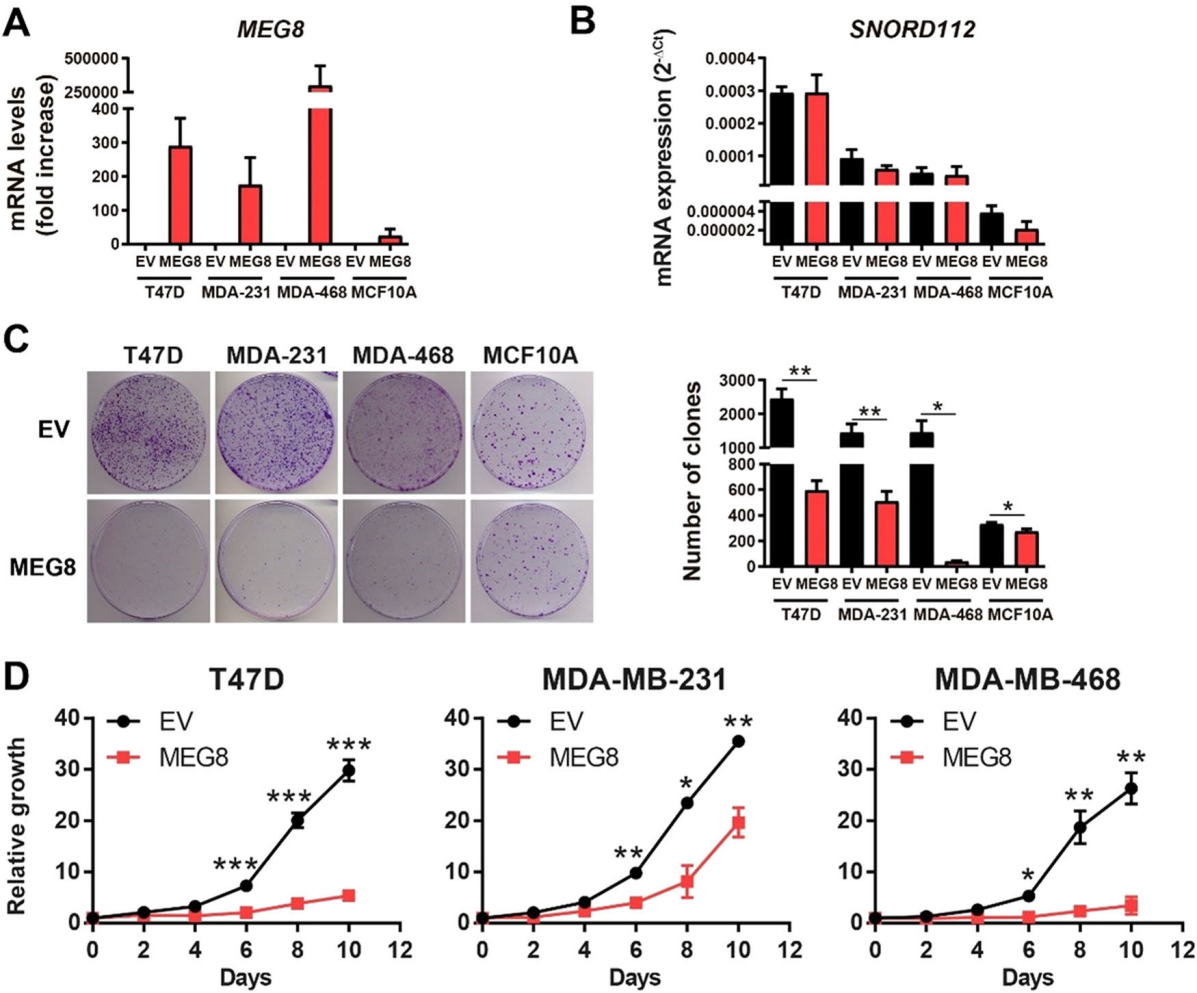
Overexpression of *MEG8* decreases the proliferative capacity of the cells in breast cancer cell lines. **A** Validation of *MEG8* overexpression by RT-qPCR. **B** Measurement of *SNORD112* expression levels by RT-qPCR in all breast control and *MEG8* cell lines. **C** Clonogenic assay of control and *MEG8* overexpressing breast cell lines. Cells were seeded at low density and after 15 days colonies were counted. Representative images are shown. **D** Growth curves of T47D, MDA-MB-231 and MDA-MB-468 control and *MEG8* cell lines. All the figures show the average of three independent experiments performed in triplicate. Data were analyzed using Student’s t-test. *p < 0.05; **p < 0.01; ***p < 0.001.

Since the levels of *MEG8* expression were low in the three breast cancer cell lines and they decrease with age and as the cell line was more tumorigenic, we overexpressed *MEG8* and an empty vector (EV) as a control to study the role of *MEG8* in breast tumors. We validated the overexpression at mRNA levels by RT-qPCR (Fig. 5A) and we corroborated that the levels of *SNORD112* were not affected by *MEG8* overexpression in all the breast cell lines used, so that overexpression was specific to *MEG8* (Fig. 5B). To study the effect of *MEG8* overexpression in the tumoral capacity of the cells, first we analysed the capability to grow in the absence of cellular contact forming colonies. In this environment, the overexpression of *MEG8* induced cells to form low number and smaller colonies than control cells in all cell lines (Fig. 5C). Next, we performed a growth curve assay, and we observed that cells that overexpress *MEG8* grew slower than control cells in all breast cancer cell lines (Fig. 5D). Thus, the overexpression of *MEG8* seems to decrease the proliferative capacity of the breast cancer cells in vitro.

**Fig. 6:**
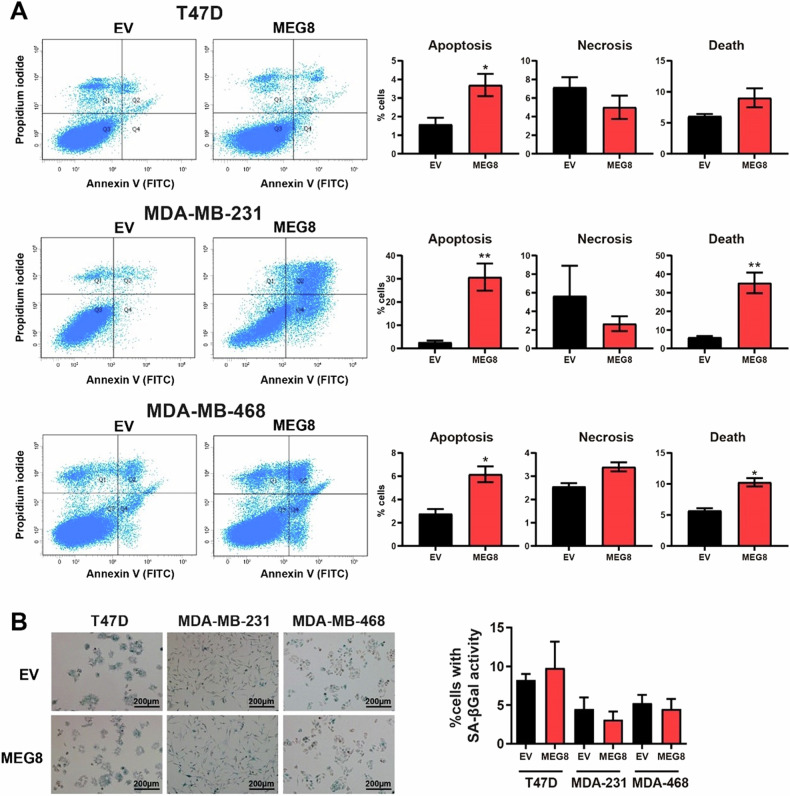
Overexpression of *MEG8* induces apoptosis but not senescence in breast cancer cell lines. **A** Measurement of apoptosis in T47D, MDA-MB-231 and MDA-MB-468 control and *MEG8* cell lines through FACS by using Annexin V and propidium iodide stainings. We consider early-apoptotic cells (Annexin positive/propidium iodide negative cells), necrotic cells (Annexin negative/propidium iodide positive cells), and apoptotic cells (Annexin positive/propidium iodide positive cells). **B** Percentage of T47D, MDA-MB-231 and MDA-MB-468 control and *MEG8* cell lines with senescence associated (SA) – β-galactosidase activity after Xgal staining. Representative images are shown. All the figures show the average of three independent experiments performed in triplicate. Data were analyzed using Student’s t-test. *p < 0.05; **p < 0.01.

Breast cancer cells that overexpress *MEG8* grew slower, therefore, we wondered if they would have activated a programmed cell death. We performed a cell death analysis in which we stained cells with both AnnexinV and PI. We found that the overexpression of *MEG8* induced an increase in the percentage of apoptotic cells in the three breast cancer cell lines studied but we did not found differences in the percentage of necrotic cells (Fig. 6A). In addition, we checked that this overexpression did not induce senescence in any of the breast cancer cell lines used since they did not have showed any differences in the acid β-galactosidase activity (Fig. 6B). Therefore, it seems that breast cancer cells with increased *MEG8* had activated a programmed cell death.

### The overexpression of *MEG8* decreases the stemness properties of breast cancer cells

**Fig. 7:**
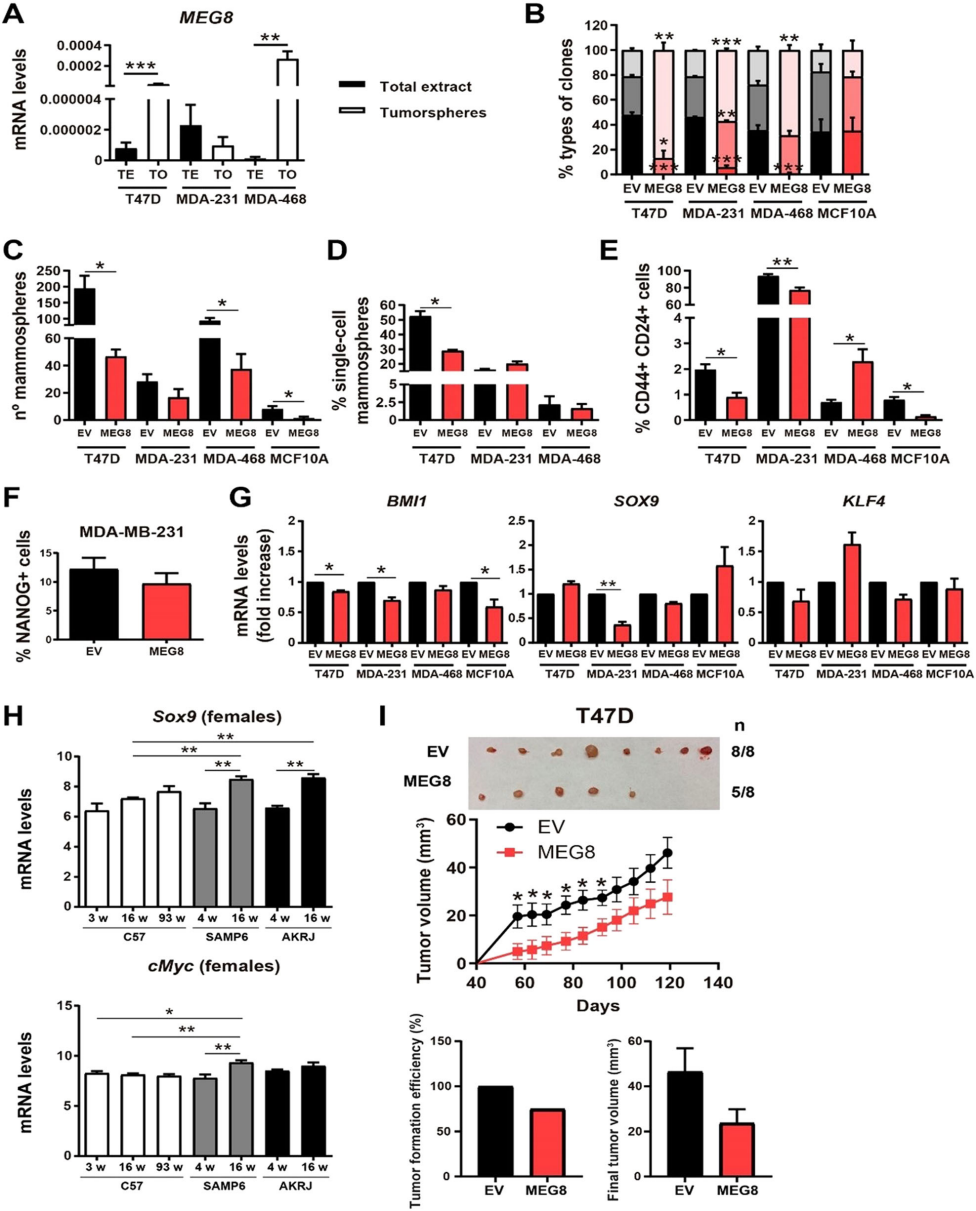
Overexpression of *MEG8* decreases the stemness properties of the cells in breast cell lines. **A** Measurement of *MEG8* expression levels by RT-qPCR in the mammospheres and the total extract of T47D, MDA-MB-231 and MDA-MB-468 cell lines. **B** Percentage of holoclones, meroclones and paraclones generated by control and *MEG8* overexpressing breast cell lines seeded at low density during 15 days. **C** Number of mammospheres formed comparing control and *MEG8* overexpressing breast cell lines. **D** Percentage of mammospheres formed from one single cell of control and *MEG8* overexpressing breast cell lines separated by FACS. **E** Quantification of the percentages of CD44 + CD24- cells in control and *MEG8* overexpressing breast cell lines by FACS. **F** Quantification of the percentage of NANOG + CD24- cells in control and *MEG8* overexpressing breast cell lines by FACS. **G** Measurement of *BMI1*, *SOX9* and *KLF4* expression levels by RT-qPCR in control and *MEG8* overexpressing breast cell lines. **H** Expression levels of *Sox9* and *cMyc* genes by a microarray analysis in the breast tissue of three different strains of mice, C57BL/6 J, SAMP6 and AKRJ. We used 3/4-, 16- or 93-weeks (w) old animals. Here, we only show the results of females (n = 3 in each condition). **I** Tumorigenicity of tumorspheres in vivo. Mammospheres from T47D and MDA-MB-468 control and *MEG8* cells were injected in nude mice (N = 8). Mice inoculated with the T47D cell line were treated with 4 mg/mL of β-estradiol. Graphs represent the tumor size (mean ± SEM). The figures show the average of three independent experiments performed in triplicate. Data were analyzed using Student’s t-test. *p < 0.05; **p < 0.01; ***p < 0.001.

To explore the effect of MEG8 overexpression on the stemness properties of the cells, we measured *MEG8* expression levels in mammospheres and total extract in T47D, MDA-MB-231 and MDA-MB-468 cell lines. We observed higher *MEG8* expression in the mammospheres of T47D and MDA-MB-468 in comparison with the total extract (Fig. 7A). We measured the phenotypes of the different clones in a clonability assay. Cells that overexpress *MEG8* formed lower number of holoclones (enriched in cancer stem cells, CSCs) and higher number of paraclones (colonies enriched in mature, non-stem cells) than control cells in all breast cancer cell lines, but we did not observe differences in MCF10A (Fig. 7B). We cultures human breast epithelial populations of cells in serum-free suspension media, forming mammospheres, which show self-renewal ability upon disaggregation and are enriched for multipotent epithelial progenitors with higher expression of CSC markers [22, 23]. We performed a mammosphere assay and we found that those cells that overexpress *MEG8* formed lower number of mammospheres than control cells in all the breast cell lines used (Fig. 7C). This result was also very evident when we seeded cells from single-cell sorting since T47D cells with *MEG8* overexpressed formed lower number of single-cell mammospheres than control cells (Fig. 7D). Breast cancer cells that overexpress *MEG8* also showed a lower proportion of CD44 + CD24- cells, which are considered tumor-initiating cells in breast tumors [23–25], except from MDA-MB-468 (Fig. 7E). In addition, since MDA-MB-231 cells have a high proportion of CD44 + CD24- cells, we measured the percentage of NANOG+ cells by FACs and we observed a decreased percentage of positive cells in MDA-MB-231 with increased *MEG8* (Fig. 7F). Additionally, we found that breast cancer cells that overexpress *MEG8* have decreased the expression levels of some stem cell markers such as *BMI1, SOX9* and *KLF4* (Fig. 7G) and in some cases also *NANOG* or *SOX2*, but not *OCT4* (Figure Supplementary S4A).

Next, we analysed the expression of some of those stem cell markers in the breast tissue of normal and premature aged mice. We found that the expression levels of *Sox9* and, partially *cMyc*, were increased with age in normal and aged mice and that the latter have higher expression levels of those stem markers than normal mice (Fig. 7H). Indeed, this difference in the expression is observed only in females, but not in males (Fig. 7H and Figure Supplementary S4B). Finally, we injected the mammospheres from T47D *MEG8* and control cells into nude mice. After 120 days, we observed that mammospheres from cells with increased *MEG8* formed tumors with lower efficiency than control cells and that those tumors formed from increased *MEG8* cells were smaller than those formed from control cells (Fig. 7I). All of these results suggest that the overexpression of *MEG8* decreases the stemness properties of breast cells.

*MEG8* has been reported to be implicated in the Notch signalling pathway, implicated in stemness [26]. Therefore, we analyzed the expression of some regulators of Notch pathway in the breast tissue of normal and premature aged mice. We found that the expression levels of *Notch1, Hes1* and *Id2* were also increased with age and that aged mice have higher expression levels of some of them (Figure Supplementary S4C). All of these results suggest that *MEG8* is implicated in the regulation of the stemness of the cells.

CSCs are responsible for the initiation of tumorigenesis, tumor progression, migration and metastasis. They divide slowly and have a greater longevity than other cell types within the tumor, which makes them resistant to conventional drugs that attack proliferating cells, producing an increase in the proportion of CSCs that generate recurrences and metastasis [22, 27–35]. Since the cells that overexpress *MEG8* had a lower proportion of CSCs, had decreased the stemness properties of the cells and had activated a programmed cell death, we studied their sensitivity or resistant to different drugs commonly used in the clinics. For that purpose, we performed different IC50 assays in our breast cancer cell lines that overexpress *MEG8*. We found that cells with increased *MEG8* were more resistant to carboplatin and 5FU treatments in all the breast cancer cell lines used. However, the overexpression of *MEG8* induced drug resistance or sensitivity to cisplatin, gemcitabine and paclitaxel depending on the cell line (Figure Supplementary S5**)**. Therefore, the overexpression of *MEG8* and its associated depletion of CSCs may only partially explain the drug response observed.

## Discussion

In this work, we described for the first time *Rian/MEG8* as a possible biomarker connecting aging and breast cancer. The mouse gene *Rian* and its human orthologous *MEG8*, are long noncoding RNAs (lncRNAs) that can be transcribed into other different long and shorts noncoding RNAs, including both miRNAs and snoRNAs. They are located in the DLK1-DIO3 cluster, which contains imprinted protein-coding genes, and several imprinted large and small noncoding RNA, such as the from maternally inherited homolog, *Rian/MEG8* [16]. Imprinted trasncripts are involved in a large amount of processes including development, growth and differentiation, including embryonic stem cell differentiation [16, 18, 36, 37], and play an essential role in human development and diseases [38]. lncRNAs, through complex molecular mechanisms, regulate gene expression and are involved in development processes, including cell and tissue-specific expression and the regulation of pluripotency and differentiation [17, 26, 36, 39]. LncRNAs deregulated expression is associated with malignant tumors [40]. Indeed, the imprinted genes in the DLK1-MEG8 locus were found silenced in several types of cancer [41].

To study aging, we used three different strains of mice as models: normal C57BL/6 J mice and premature aged mice (specifically SAMP6/TaHsd and AKRJ strains), which present a reduced lifespan of 300 days compared to 900 days for C57 mice. SAMP6 (Senescence Accelerated Mouse Prone 6) mice show accelerated aging due to a senile osteoporosis characterized by a diminished bone formation and a paucity of osteoblast progenitor cells [19] whereas AKRJ mice present a high leukemia incidence (60-90%) [20]. First of all, we performed a microarray from breast tissue from old and young mice from both sexes of the three strains of mice. When analyzing the variations in those genes differentially expressed between young and old mice in each strain, we found that *Rian* was the gene with most significant and pronounced changes. Indeed, *Rian* was the only gene that appears when we overlap the differentially expressed genes of the three strains together including old and young mice. Therefore, *Rian* was the only gene that had a different expression pattern in breast tissue in the three strains of mice analyzed, showing a decrease in its expression with age. In addition, we found that *Rian* expression levels were higher in breast tissues of premature aged mice in comparison with those of C57 mice.

However, the role of *Rian/MEG8* in aging and cellular senescence remains largely unknown. In this work, we explored the role of *Rian/MEG8* in these processes trying to identify the connexion between aging, cellular senescence and cancer in the breast tissue.

Our results also showed that this differential expression of *Rian* in the microarray was gene-specific since other genes of the DLK1-DIO3 cluster did not show differences in expression. Furthermore, this pattern of expression of *Rian* was independent on the sex. We also observed that the expression levels of the progesterone receptor (*Pgr*) and *Her2* genes increased with age in the three different strains, especially in females, whereas the expression levels of the estrogen receptor *Esr1* only increased in normal females. When we analyzed its human homologous *MEG8* by using human blood samples from a cohort of patients with different age, we found that *MEG8* expression also decreased with age since adult patients presented lower levels than teen or child patients. In addition, the levels of *SNORD112* and *SNORD113*, two different C/D box snoRNAs located very close to *MEG8*, also decreased with age, suggesting that this region is clearly involved in aging.

Since *Rian/MEG8* expression decrease with age and is high in mice that age prematurely, we wonder if this gene could act as an antagonistically pleiotropic gene during aging: suppressing the development of cancer early in life and driving age-related pathologies such as cancer late in life. Breast cancer constitutes the most common neoplasm among women, the leading cause of death from cancer in women worldwide [10–12], and one of the risk factors that increase the probability of breast cancer is an age over 50 years [13]. Therefore, we focused on elucidating the role of *MEG8* in the connection of aging and breast cancer.

First, we explored the role of *MEG8* in normal cells and we found that the overexpression of *MEG8* induces senescence in both mouse embryonic fibroblasts and in human non-tumorigenic cells from breast tissue. Then, we found that the overexpression of *MEG8* decreases the proliferative capacity of breast cancer cells both by activating a programmed cell death program such as apoptosis. Indeed, it has been reported that enhanced *MEG8* expression suppressed the proliferation and migration ability of vascular smooth muscle cells by inducing apoptosis [42] and the expression of pro-fribrogenic and proliferation genes in activated hepatic stellate cells [26]. We also observed that the levels of *MEG8* decreased as the cell line was more tumorigenic or aggressive, corresponding to basal/triple negative tumors, whereas the non-tumorigenic breast cell line MCF10A had the highest levels. This correlated with a decreased expression of *MEG8* observed in breast tumors in comparison with normal tissue from breast cancer patients of the TCGA database and the fact that the cluster DLK1-MEG8 has been found silence in several types of cancer [41]. In addition, breast tumors presented a higher proportion of shallow or deep deletions in the gene. Therefore, these results could correspond with an antagonistically pleiotropic behavior, in which *MEG8* levels decrease as the tumor becomes more aggressive, losing its suppressive effect observed in normal cells when the levels of *MEG8* are high.

Tumors are very heterogeneous entities composed of different types of cells, among which are cancer stem cells (CSCs), which are responsible for the initiation of tumorigenesis, tumor progression, migration and metastasis. CSCs divide slowly and have a greater longevity than other cell types, which makes them resistant to conventional drugs that attack proliferating cells, producing an increase in the proportion of CSCs that generate recurrences and metastasis [22, 27–35]. In addition, tumor cells exhibit plasticity, so the different cell subpopulations of a tumor can pass from one stage to another and new CSCs are generated from mature tumor cells through dedifferentiation. This tumor evolution may lead to increased resistance to conventional therapies and may be responsible for recurrences and residual disease [43]. We found that the overexpression of *MEG8* decreases the number of CSCs and the stemness properties of breast cancer cell lines. We also found that *MEG8* overexpression decreased the expression of some stem cell markers, especially *SOX9* and *BMI1*. SOX9 is overexpressed in a wide range of human tumors, where it contributes to proliferation, tumor progression and malignancy [44, 45]. Besides, SOX9 has been reported as a novel regulator of senescence in vitro, and its activation allows to escape senescence [44]. Indeed, SOX9 constitutes an important regulator of breast cancer survival and metastasis [46, 47]. LncRNAs are associated with stemness and are involved in the maintenance of pluripotency. It has been shown that LncRNAs regulate transcription factors levels, or participate in the reprogramming process [48]. All of this could explain the inverse correlation between *MEG8* and *SOX9*, since when *MEG8* is overexpressed it shows a tumor suppressor role that could not be bypassed by the low levels of SOX9. However, when cells lose the expression of *MEG8*, SOX9 activity and its downstream target BMI1, proliferation is promoted, bypassing senescence and facilitating tumor formation and progression [44]. In addition, *MEG8* seems to regulate other stemness-related pathways in other cells types, since *MEG8* inhibits the Notch signaling pathway and suppressed the epithelial-mesenchymal transition of hepatocytes [26]. We also observed that the expression of some Notch pathway genes increased with age in our microarray study in an opposite way than *MEG8*. Regarding resistance to conventional therapies, we found that cells that overexpress *MEG8* were more resistant to some treatments and more sensible to others in vitro, so the overexpression of *MEG8* and its associated depletion of CSCs may only partially explain the drug response observed.

## Conclusions

Senescent cells accumulate with age, therefore *MEG8* could be the result of the antagonistic pleiotropic theory, in which cellular senescence is beneficial early in life as a result of a tumor suppression mechanism because *MEG8* is increased and causing few breast tumors with aging. Conversely, this effect could be detrimental later in life as a result of aging and cancer, when *MEG8* is reduced and lose its tumor suppressor role. Therefore, *MEG8* could be the link of senescence, aging and breast cancer, and this effect could be mediated by SOX9. This antagonistic theory of aging is supported by other studies and genes, for example the genetic overactivation of insulin-like growth factor (IGF-1) signaling improves cardiac function in young mice but causes premature heart failure during aging. Indeed, its inhibition avoids age-related heart failure in old mice, extending their lifespan [49]. In human, different studies have also proposed that extremely high levels of IGF-1 may be detrimental in aging, while extremely low levels are associated with both benefits and detriments. Therefore, IGF-1 is generally associated with protection from disease in younger individuals and with increased risk for morbidity in older individuals [50]. The evolutionary theory of aging suggests that aging requires mutations with age-specific effects and two nonexclusive mechanisms have been proposed. The first is the already mentioned antagonistic pleiotropic theory, in which mutations that cause aging may have early-life beneficial effects that gradually become deleterious with age and accumulate. In the other, the mutation accumulation theory, mutations are neutral early in life and only late-life deleterious mutations have effects in aging [51, 52]. This not seems to be the case of the effect observed here for *MEG8*, in which the antagonistic pleiotropic theory fix better. However, although both theories support for each other, more studies that explain better the behavior of aging are needed.

## Materials and methods

### Animal cohorts

All animal experiments were conducted according to the experimental protocol approved by the IBIS and HUVR Institutional Animal Care and Use Committee (0309-N-15). Three different strains of mice were used: C57BL/6 J, SAMP6/TaHsd, and AKR/J mice (from Jackson). The three strains of mice were kept in identical controlled conditions and sacrificed at 3-4 weeks or 16 weeks, and at 93 weeks for C57BL/6 J mice, selecting three females and three males from each condition to collect breast tissue samples. Randomization was not used. Blood samples were collected from healthy children aged 6-10 years. Signed informed consent to participate was obtained from the parents or legal guardians. The project was approved by the Research and Ethics Committee of La Rioja (Spain) (CEICLAR PI 289).

### Microarray analysis

We extracted and purified total RNA from mouse tissue samples using the miRNeasy Mini Kit (Qiagen) in accordance with the manufacturer’s guidelines. RNA integrity was assessed using the RIN parameter with the Agilent 2100 Bioanalyzer System. Microarray experiments were conducted using the Affymetrix GeneChip® Mouse Gene 2.0 ST Array. The investigator conducting the measurements was blinded to the group assignments.

Data analysis was performed in R. Raw microarray data were imported using the read.celfiles function from the Bioconductor *oligo* package. Preprocessing steps (background correction, quantile normalization, and summarization) were performed with the rma function from the same package. Principal Component Analysis (PCA) was conducted on normalized intensity data using the prcomp function. Differential expression analyses between young and old samples were carried out across all strains using a linear model with the limFit and eBayes functions from the *limma* package, applying an adjusted p-value threshold of 0.05. Similarly, strain-specific differential expression was determined, and overlapping genes were visualized using the *VennDiagram* package. Functional enrichment analysis was conducted with the goana function from *limma*.

### Cell culture

Mouse embryonic fibroblasts (MEFs) were generated and subjected to the 3T3 protocol as previously reported. The MCF10A, MDA-MB-468, T47D and MDA-MB-231 cell lines were obtained from the ECACC repository without further authentication. All cell lines were confirmed to be mycoplasma-free and cultured in DMEM (Sigma) supplemented with 10% fetal bovine serum (FBS, Gibco), penicillin, streptomycin, and fungizone (Sigma).

### Transfection and plasmids

MEFs were transfected following a previously described protocol, while breast cell lines were transfected at subconfluency using the TransIT-X2 reagent (Mirus) per the manufacturer’s instructions. Cells were seeded into 10-cm plates with selection media containing puromycin (0.25–0.5 µg/mL) 48 hours post-transfection. The plasmids used included the pBabe-puro-empty vector (EV) and pRetroG-CMV-MEG8 (Applied Biological Materials, RV2175741).

### RT-qPCR

Total RNA was extracted from cell lines using the ReliaPrep™ RNA Tissue Miniprep System (Promega). Reverse transcription was performed on 3 µg RNA using the High Capacity cDNA Reverse Transcription Kit (Life Technologies). The PCR mixture (10 µL) included 2 µL cDNA, 2.5 µL water, 5 µL GoTaq® Probe qPCR Master Mix (Promega), and 0.5 µL TaqMan Assay (Applied Biosystems). Endogenous controls were Gapdh (Mm99999915_g1) and GAPDH (Hs03929097_g1). Rian (Mm01325842_g1); MEG8 (Hs00419701_m1); SNORD112 (Hs03298810_s1); BMI1 (Hs00995536_m1); SNORD113-4 (Hs03299143_sH); p21CIP1 (Mm04205640_g1); p16INK4a (Mm00494449_m1); p15INK4b (Mm00483241_m1); OCT4 (Hs00999632_g1); p19ARF (Mm00486943_m1); KLF4 (Hs00358836_m1); NANOG (Hs04260366_g1); SOX2 (Hs01053049_s1); SOX9 (Hs01001343_g1); and c-MYC (Hs00153408_m1). The investigator was blinded to group assignments during analysis.

### Growth curve assay

Proliferation was assessed by seeding 1 × 10^4^ T47D cells or 5 × 10³ MDA-MB-468 and MDA-MB-231 cells in 6-well plates. After 24 hours (day 0), cells were fixed with 0.5% glutaraldehyde. Data points were collected every 48 hours for up to 15 days. Plates were stained with 1% crystal violet, and relative cell numbers were quantified by measuring absorbance at 595 nm.

### Cell death analysis

Cell death was assessed using the FITC Annexin V Apoptosis Detection Kit with propidium iodide (Immunostep) according to the manufacturer’s protocol. Apoptosis levels were measured by flow cytometry on a Canto II flow cytometer, and data were analyzed using BD FACS Diva and FlowJo software.

### Clonogenic assay

To assess clonogenic potential, 5 × 10³ cells were plated in 10-cm dishes in triplicate. After 15 days, cells were fixed with 0.5% glutaraldehyde and stained with 1% crystal violet. Colonies were counted, and clone types were categorized.

### Tumorsphere assay

Tumorsphere formation was evaluated by seeding 5 × 10³ cells in triplicate into 24-well Ultra-Low Attachment Plates (Costar) containing 1 mL of MammoCult basal medium (Stemcell Technologies). Media was supplemented with 10% proliferation supplement, 0.48 μg/mL hydrocortisone, 4 μg/mL heparin, penicillin, and streptomycin. Tumorspheres were counted after 5–10 days using an Olympus IX-71 inverted microscope.

### Single-cell tumorsphere assay

Single-cell tumorsphere formation was assessed by sorting individual cells into 96-well Ultra-Low Attachment Plates using a FACS Jazz flow cytometer (BD Biosciences). Plates contained 1 mL MammoCult basal medium with supplements as described above. After 30 days, tumorsphere numbers were quantified using an Olympus IX-71 inverted microscope.

### Fluorescence-activated cell sorting (FACS) analysis

For FACS, 1 × 10^6^ cells were trypsinized and resuspended in PBS with 2% FBS and 5 mM EDTA. Cells were blocked with 12.5 μL of human blocking reagent (Miltenyi Biotec) at 4°C for 10 minutes, followed by incubation with anti-CD44-FITC (Miltenyi Biotec #130-113-331) and anti-CD24-PE (Miltenyi Biotec #130-095-953) for 30 minutes at 4°C. After two PBS washes, cells were resuspended in 500 μL of buffer for analysis on a FACS Canto II cytometer. For nuclear NANOG staining, cells were fixed in 4% paraformaldehyde, permeabilized with 0.5% Triton X-100, and incubated with anti-NANOG (Santa Cruz sc-293121), followed by Alexa 488-conjugated secondary antibody (Thermo Fisher A11029). Analysis was performed on a FACS Canto II cytometer. All experiments were repeated independently three times, with triplicates.

### β-galactosidase (X-Gal) staining

Cells were washed with PBS, fixed in 0.5% glutaraldehyde, and incubated in X-Gal staining solution (1.25 mg X-Gal, 5 mM potassium ferricyanide, and 5 mM potassium ferrocyanide trihydrate in PBS with 1 mM MgCl_2_ at pH 5.5) at 37°C. Staining was visible after 4 hours, and the percentage of SA-βGal-positive cells was quantified.

### Xenograft in nude mice

Tumorigenicity was evaluated by injecting 8 × 10^6^ T47D cells, 5 × 10^6^ MDA-MB-468 cells, or 4 × 10^6^ MDA-MB-231 cells subcutaneously into the right flanks of 4-week-old female athymic nude mice. Cells were mixed with 50 μL of Matrigel (Corning) prior to injection. Tumors were monitored weekly, and mice were euthanized 60–70 days later, depending on the cell line. Tumorsphere-derived cells were similarly injected following disaggregation. For T47D xenografts, mice received 4 mg/mL β-estradiol (Sigma) in drinking water. Tumor volume was measured with calipers. All procedures followed the protocols approved by the IBIS and HUVR Institutional Animal Care and Use Committee (0309-N-15).

### Protein isolation and Western blot analysis

Protein extraction and Western blotting were performed as described previously. Primary antibodies included anti-PARP, anti-Caspase 9, anti-Caspase 3, anti-BAX, anti-Bcl-xl, anti-Phospho H2A-X Ser139, anti-SOX9, anti-c-MYC, and anti-α-tubulin (used as a loading control). Secondary antibodies were horseradish peroxidase-labeled rabbit anti-mouse and goat anti-rabbit (Abcam). Proteins were detected with an ECL system (Amersham) and imaged using a Bio-Rad Chemidoc Touch system.

### Cytotoxic assay

Cytotoxicity studies involved seeding 30,000 cells per well in 96-well plates. After 24 hours, cells were treated with varying concentrations of cisplatin, carboplatin, 5-FU, gemcitabine, or paclitaxel. Treatments lasted 96 hours, after which cells were fixed with 0.5% glutaraldehyde and stained with 1% crystal violet. The absorbance of dissolved crystal violet (in 20% acetic acid) was measured at 595 nm. The IC50 values were determined using GraphPad Prism software.

### Public databases of clinical samples

Clinical and genomic data were retrieved from publicly accessible platforms, including the R2 Genomics Analysis and Visualization Platform, the TCGA Research Network, and cBioPortal.

### Statistical analysis

Statistical analyses were conducted using GraphPad Prism (version 6.01). Data from control (EV) and MEG8-overexpressing samples were compared using unpaired Student’s t-tests or Welch’s t-tests, where appropriate. Experiments were conducted independently at least three times in triplicate. *P* values < 0.05 were considered statistically significant and are represented as *p* < 0.05 (*), p < 0.01 (**), and p < 0.001 (***).

## Supplementary information

SUPPLEMENTARY FIGURE LEGENDS

Figure S1

Figure S2

Figure S3

Figure S4

Figure S5

Original Data

## Data Availability

Data from publicly available clinical and genomic information were obtained from the R2 Genomics analysis and visualization platform (https://hgserver1.amc.nl/cgi-bin/r2/main.cgi), the TCGA Research Network (https://cancergenome.nih.gov/) and the cBioPortal (http://www.cbioportal.org/).
