# Supplementary material for: MEG8 as an antagonistic pleiotropic mechanism in breast cancer
Source: Cell Death Discov. 2024 Dec 20;10:509. doi: 10.1038/s41420-024-02272-0 (PMC11662018; doi:10.1038/s41420-024-02272-0)
Supplement: Supplementary file 1 — SUPPLEMENTARY FIGURE LEGENDS [file 41420_2024_2272_MOESM1_ESM.docx]

**SUPPLEMENTARY FIGURE LEGENDS**

**Figure Supplementary S1: *Rian* is located in the DLK1-DIO3 cluster.** Scheme of *Rian* gene **(A)** and the DLK1-DIO3 cluster **(B)**. The three imprinted protein-coding genes that are paternally expressed (DLK1, RTL1 and DIO3) are highlighted in blue, whereas the maternally imprinted noncoding RNA genes are highlighted in red.

**Figure Supplementary S2: The expression levels of different hormone receptors increases with age.** Expression levels of *Esr1, Pgr* and *Her2* by a microarray analysis in the breast tissue of three different strains of mice, C57BL/6J, SAMP6 and AKRJ. We used 3/4-, 16- or 93-weeks (w) old animals. Here, we independently show the results of females and males (n=3 in each condition). Data was analyzed using Student’s t-test. * p < 0.05, ** p < 0.01, *** p < 0.001.

**Figure Supplementary S3: Overexpression of *MEG8* in MCF10A cells does not induce apoptosis.** Measurement of the levels of different apoptotic markers by western blot analysis. The figure shows the average of three independent experiments. Fig also shows uncropped blots.

**Figure Supplementary S4: *MEG8* seems to regulate the expression of stem cell markers. A)** Measurement of *NANOG*, *SOX2* and *OCT4* expression levels by RT-qPCR in control and *MEG8* overexpressing breast cell lines. **B)** Expression levels of *Sox9* and *cMyc* genes by a microarray analysis in the breast tissue of three different strains of mice, C57BL/6J, SAMP6 and AKRJ. We used 3/4-, 16- or 93-weeks (w) old animals. Here, we only show the results of males (n=3 in each condition). **C)** Expression levels of *Notch1, Hes1* and *Id2* genes by a microarray analysis in the breast tissue of three different strains of mice, C57BL/6J, SAMP6 and AKRJ. We used 3/4-, 16- or 93-weeks (w) old animals. Here, we only show the results of females (n=3 in each condition). The figures show the average of three independent experiments performed in triplicate. Data were analyzed using Student’s t-test. *, p < 0.05; **, p < 0.01.

**Figure Supplementary S5: Overexpression of *MEG8* induces different drug resistance/sensitivity depending on the cell line. A-E)** Curves showing the IC50 of control and *MEG8* cells after cisplatin **(A)**, carboplatin **(B)**, 5FU **(C)**, gemcitabine **(D)** and paclitaxel **(E)** treatment during 96 hours in breast cancer cell lines**.** The mean of a minimum of 3 independent experiments performed in triplicate ± standard deviation is represented. * p < 0.05, ** p < 0.01.
