# Supplementary figures and images for: MEG8 as an antagonistic pleiotropic mechanism in breast cancer
Source: Cell Death Discov. 2024 Dec 20;10:509. doi: 10.1038/s41420-024-02272-0 (PMC11662018; doi:10.1038/s41420-024-02272-0)

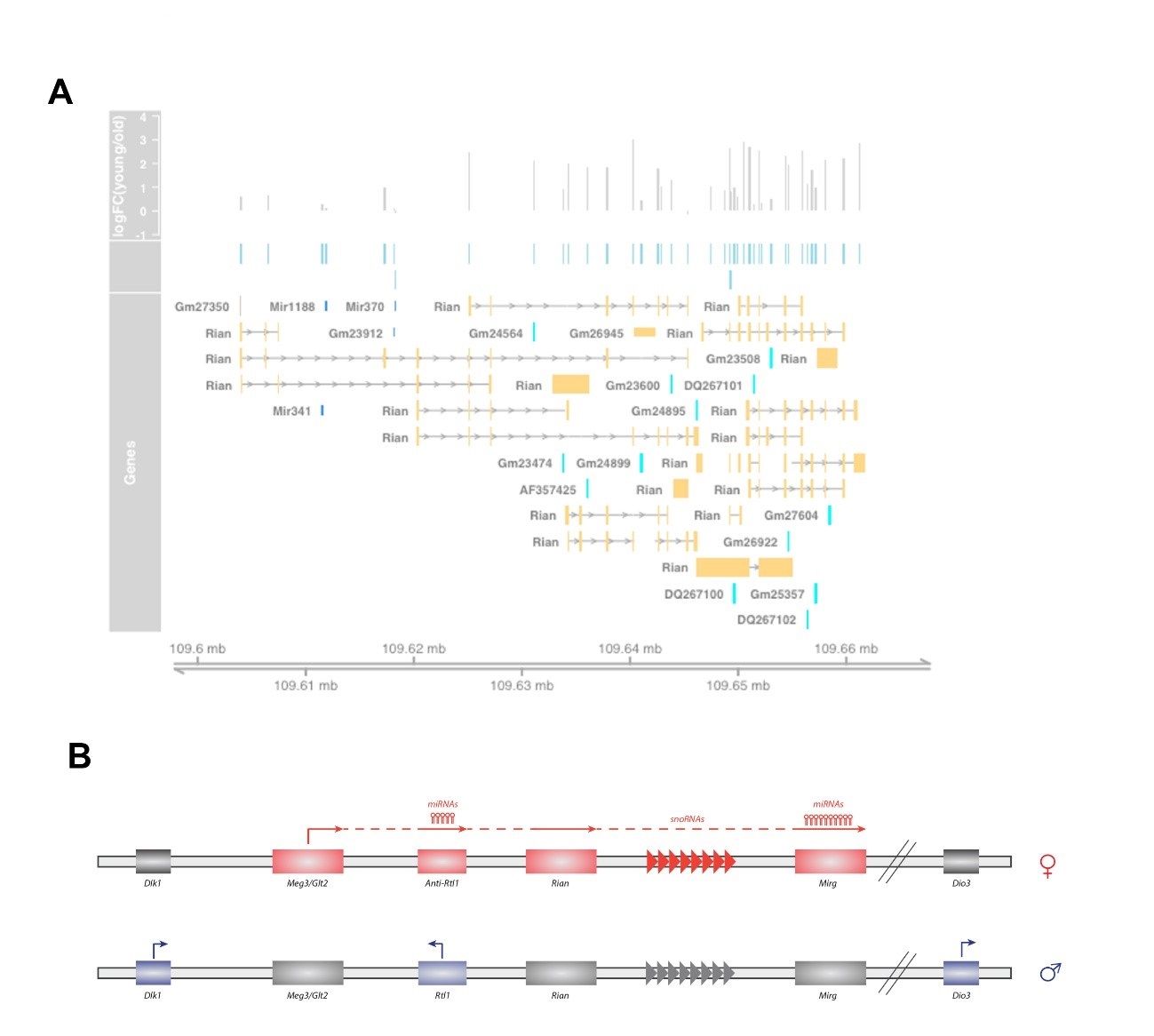

Supplement: Supplementary file 2 — Figure S1 [file 41420_2024_2272_MOESM2_ESM.jpg]

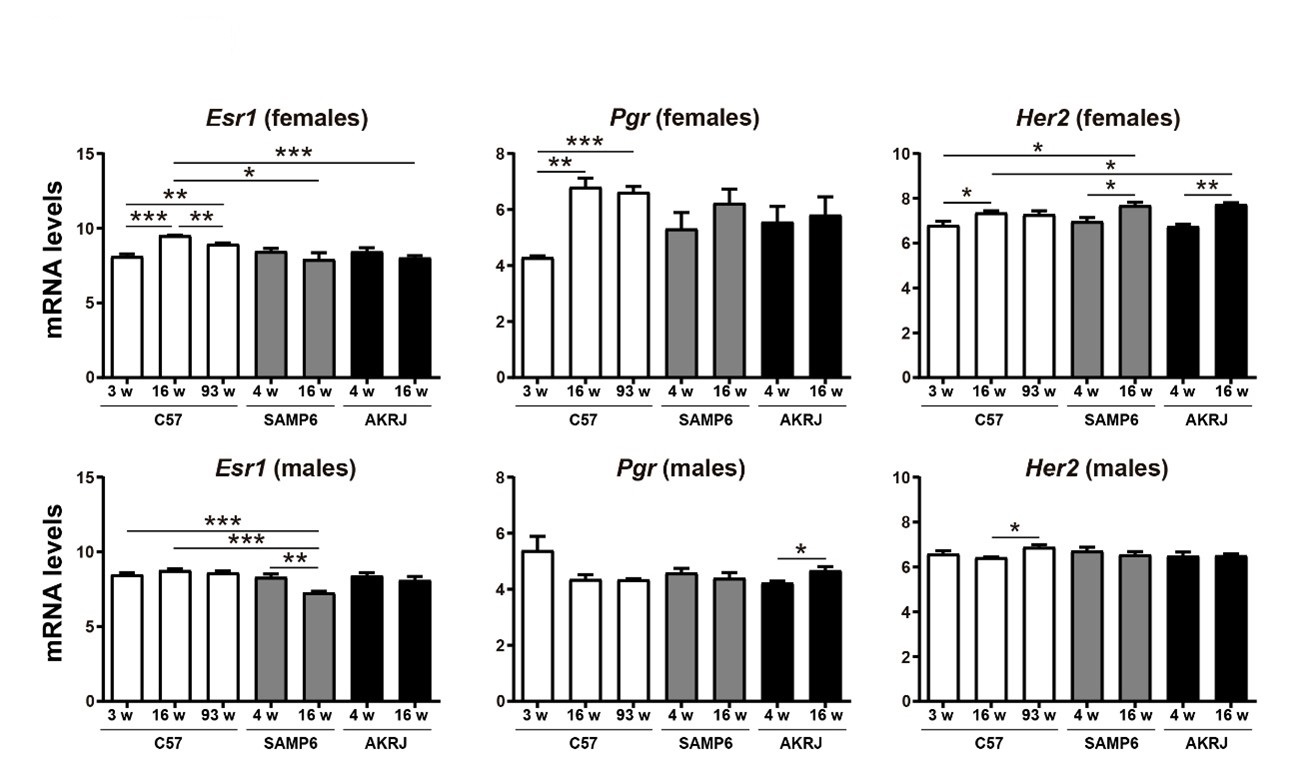

Supplement: Supplementary file 3 — Figure S2 [file 41420_2024_2272_MOESM3_ESM.jpg]

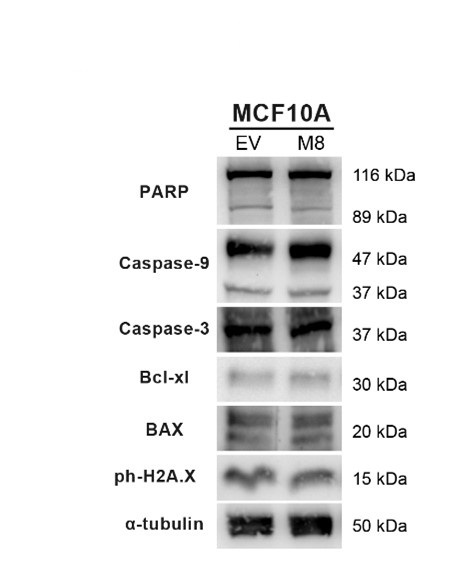

Supplement: Supplementary file 4 — Figure S3 [file 41420_2024_2272_MOESM4_ESM.jpg]

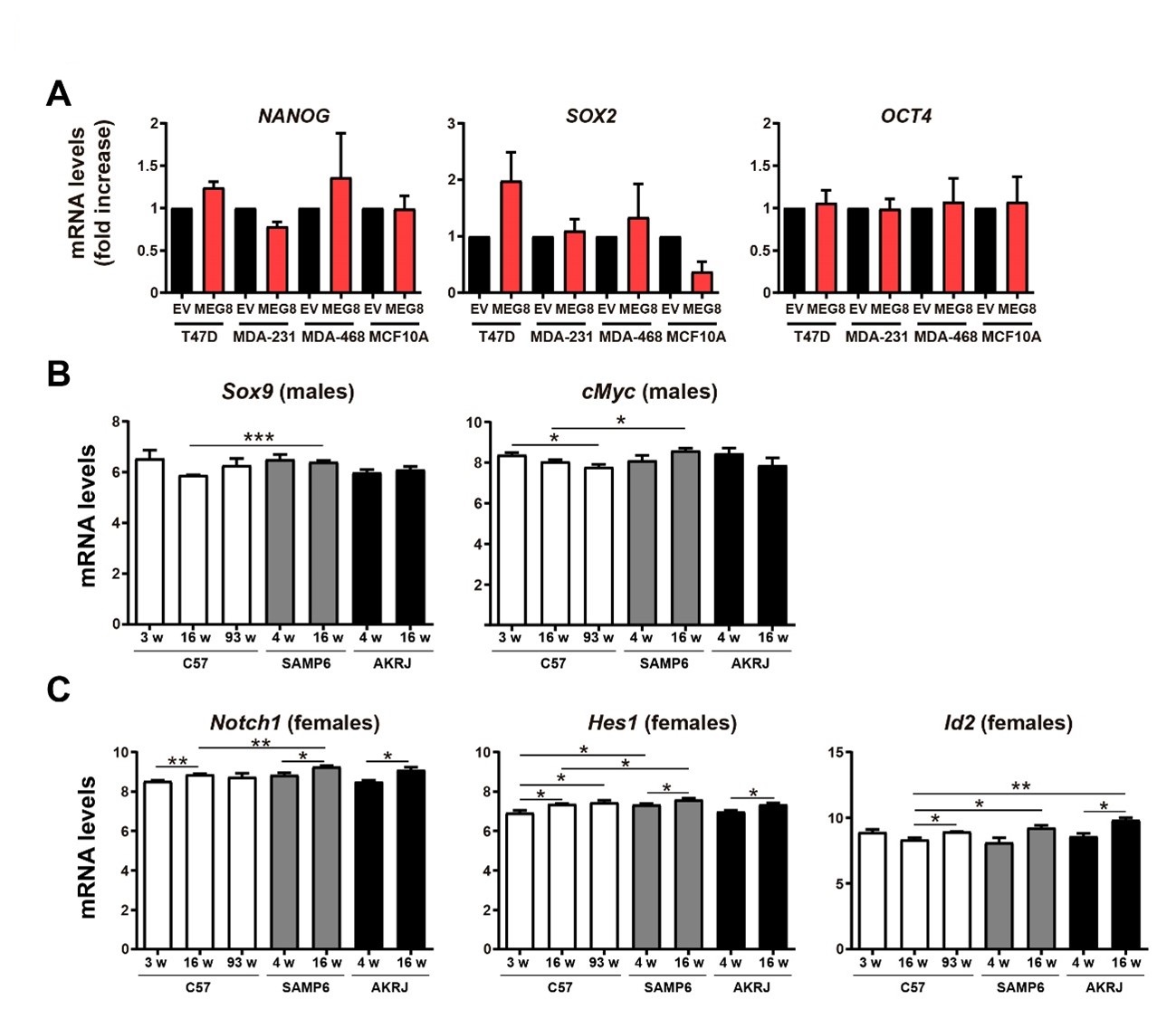

Supplement: Supplementary file 5 — Figure S4 [file 41420_2024_2272_MOESM5_ESM.jpg]

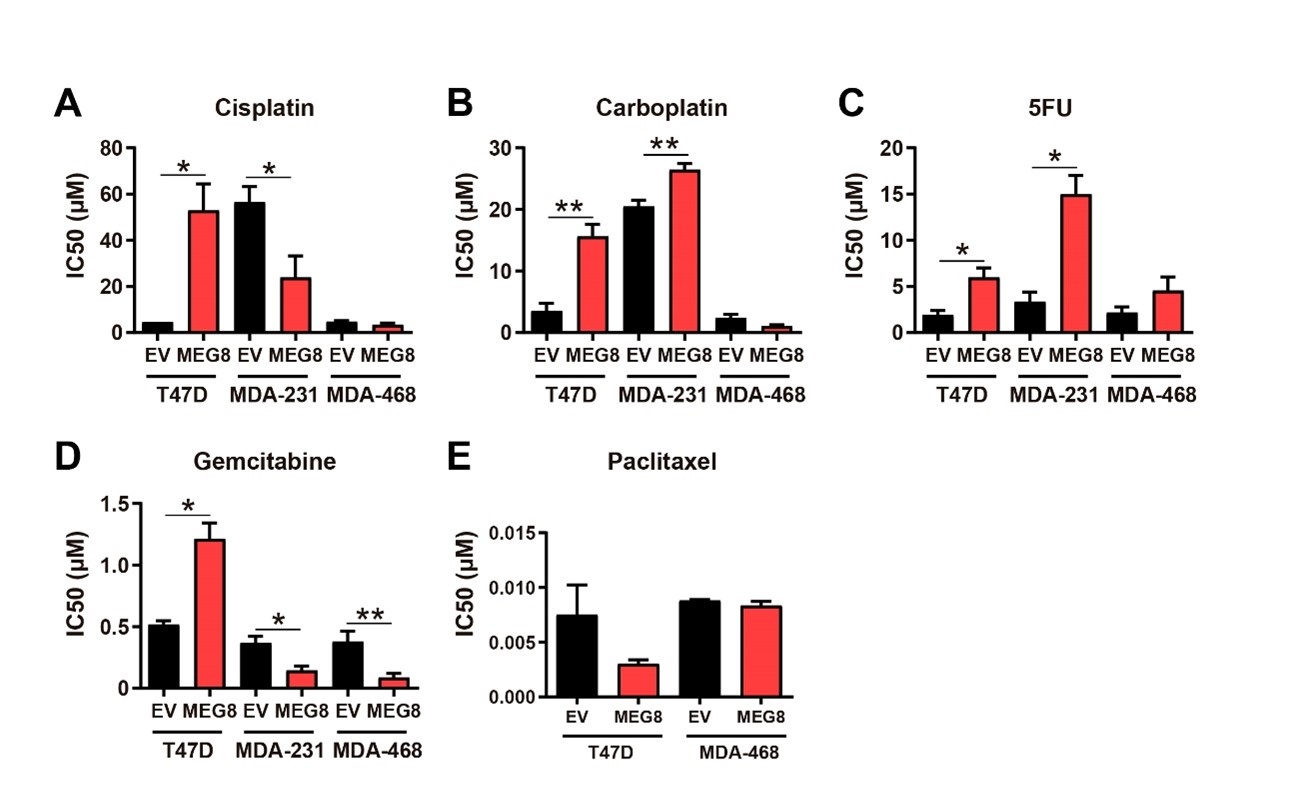

Supplement: Supplementary file 6 — Figure S5 [file 41420_2024_2272_MOESM6_ESM.jpg]

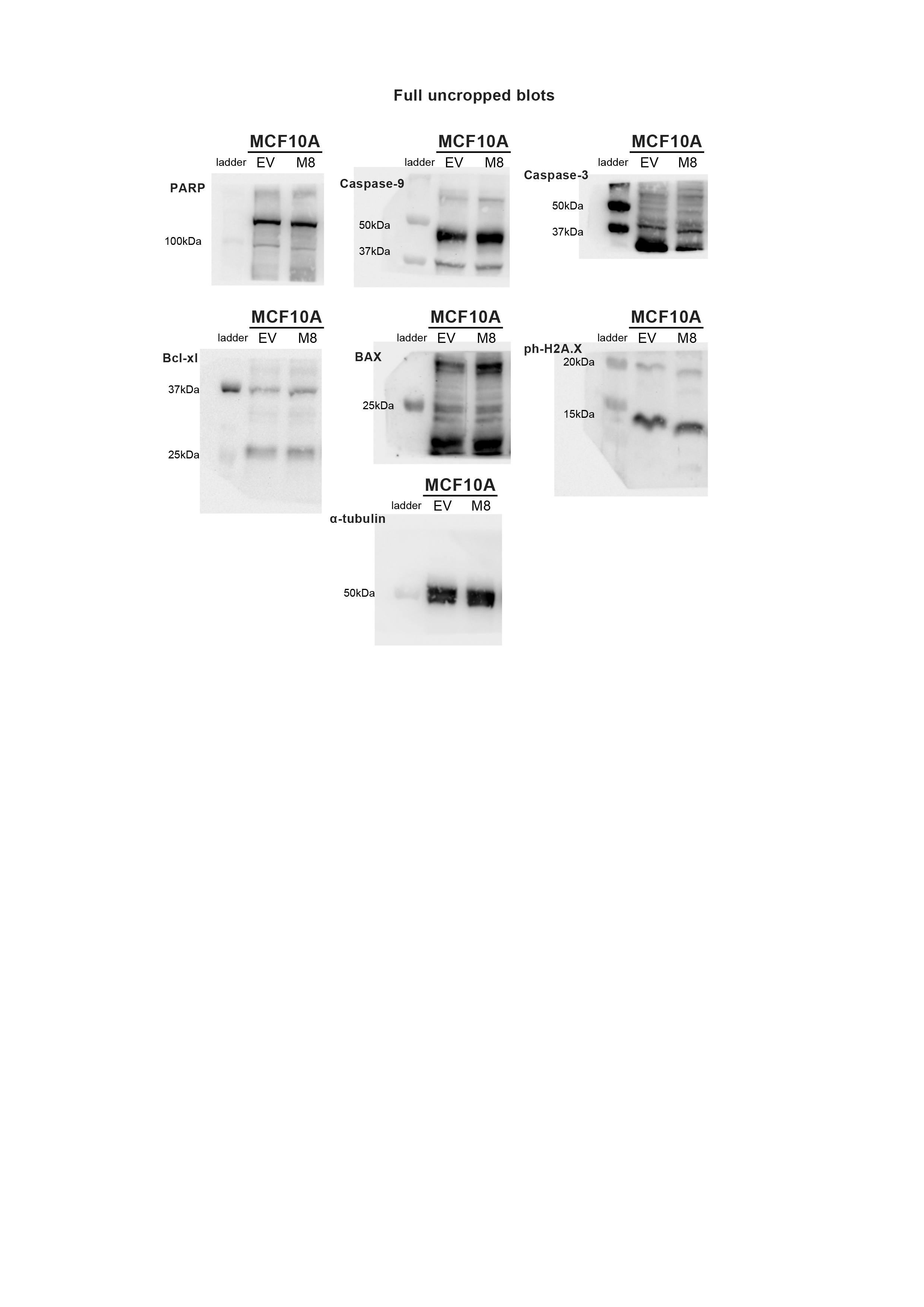

Supplement: Supplementary file 7 — Original Data [file 41420_2024_2272_MOESM7_ESM.png]
